# Differential patterns of cross-protection against antigenically distinct variants in small animal models of SARS-CoV-2 infection

**DOI:** 10.1038/s44298-025-00125-w

**Published:** 2025-06-04

**Authors:** Prabhuanand Selvaraj, Charles B. Stauft, Shufeng Liu, Kotou Sangare, Tony T. Wang

**Affiliations:** Division of Viral Products, Center for Biologics Evaluation and Research, Food and Drug Administration, Silver Spring, MD USA

**Keywords:** Live attenuated vaccines, SARS-CoV-2

## Abstract

Continuous evolution of severe acute respiratory syndrome coronavirus 2 (SARS-CoV-2) will likely necessitate periodic updates of vaccine composition. Based on a series of studies carried out in human ACE2 transgenic mice (K18-hACE2) and Syrian hamsters, we show that immunity at the respiratory tract, acquired through either previous infection or vaccination with an in-house live attenuated virus, offers protection against antigenically distinct variants in the absence of variant spike-specific neutralizing antibodies. Interestingly, immunity acquired through infection of a modern variant (XBB.1.5) was insufficient in preventing brain infection by the ancestral virus (WA1/2020) in K18-hACE2 mice. Similarly, previous infection with WA1/2020 did not protect against brain infection by XBB.1.5. Our results highlight the importance of immune components other than neutralizing antibodies in maintaining protection against new variants in the respiratory tract.

## Introduction

Initial vaccination with two mRNA vaccines against SARS-CoV-2 provided up to 95% efficacy against severe disease outcomes^1,2^. The emergence of new SARS-CoV-2 variants, however, has significantly reduced vaccine effectiveness against symptomatic COVID-19. Since 2021, various Omicron sub-lineages have rapidly replaced previously circulating variants worldwide^3^. Despite laboratory evidence of attenuated pathogenicity, increased transmissibility of Omicron sub-lineages led to large numbers of hospitalizations and deaths^4–6^. New studies consistently demonstrate a reduction in circulating neutralizing antibody (nAb) titers against emerging Omicron subvariants among those who received either the ancestral or bivalent (Wuhan-1 and BA.4/5) mRNA vaccines^7–10^. For this reason, regulatory agencies from both the United States and Europe recommended an updated monovalent vaccine composition in 2023. The 2024–2025 COVID-19 vaccines by Moderna and Pfizer-BioNTech are based on the KP. 2 variant, whereas the Novavax COVID-19 Vaccine (2024–2025 Formula) contains the spike protein of the JN.1 variant.

An intriguing question associated with a simplified vaccination regimen is whether it will protect against an earlier variant. Although new SARS-CoV-2 variants appear to have replaced the older ones, data from wastewater monitoring suggest that older cryptic lineages may exist for extended periods^11^. Additionally, newborns will continue adding to the population who are immune naïve to established SARS-CoV-2 variants. In this study, we set out to test the cross-protectivity offered by natural infection and vaccination with a live-attenuated vaccine candidate (LAV) against ancestral and contemporary SARS-CoV-2 variants in mice and Syrian hamsters. Our results show sustained protection in the respiratory tracts in K18-hACE2 mice, but not in the brain. Vaccination with WA1/2020 (WA1) and BA.5 specific LAV candidates resulted in variant-specific as well as cross-reactive humoral immunity and protection in Syrian hamsters against EG.5.1 challenge. These results have significant implications in pre-clinical evaluation of future vaccines.

## Results

### Cross protection of convalescent K18-hACE2 mice in the respiratory tract but not in the brain in the absence of circulating nAbs

**Fig. 1:**
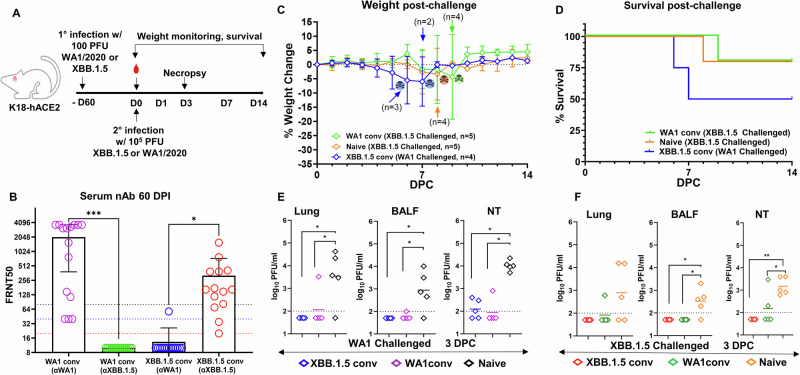
Convalescent K18-hACE2 mice are protected against rechallenge with a homologous or heterologous virus at the respiratory tract. **A** Overall design of the study. **B** Serum collected at day 60 after the first low-dose infection (before rechallenging) tested for serum nAb against WA1 & XBB.1.5 in WA1 convalescent group (*n* = 15 mice) or XBB.1.5 convalescent group (*n* = 14 mice) by FRNT_50_ assay (paired t test; error bars indicate standard deviation; *p = 0.012, ***p = 0.0003). **C** Weight loss profile and survival (**D**) of convalescent mice (WA1 conv or XBB.1.5 conv) after a challenge with 10^5^ PFU of WA1 or XBB.1.5. Three groups of animals were monitored for this part of the study as indicated. Death and the numbers of remaining animals are indicated in (**C**). **E**, **F** Lungs (*p < 0.05), BALF (*p < 0.05) and nasal turbinates (*p < 0.05, **p = 0.002) were harvested at 3 DPC and measured for infectious viral titer by plaque forming assay analyzed using one-way analysis of variance (ANOVA) (*n* = 5 animals each group).

The K18-hACE2 mice are highly susceptible to SARS-CoV-2 with a lethal phenotype to certain variants^12–19^. We have previously reported that more than 80% of K18-hACE2 mice succumbed to infection by 10^4^ plaque-forming units (PFU) WA1 isolate^20^. To derive convalescent animals, we infected K18-hACE2 mice with 100 PFU of WA1 or the XBB.1.5 variant (Fig. 1A) (*n* = 15 per group). All animals except one infected with XBB.1.5 survived the infection. At 60 days post infection (DPI), serum nAb titers were determined by 50% focus reduction neutralization test (FRNT_50_). All animals seroconverted and displayed significant nAb titers against the homotypic variant i.e., WA1 convalescent (anti [α]-WA1) [geometric mean titers (GMT) 787.2, interquartile range (IQR) 3631, *p* = 0.0003] and XBB.1.5 convalescent (α-XBB.1.5) [GMT 174.7, IQR 1534, *p* = 0.012] (Fig. 1B). By contrast, nAb titers of the WA1-convalescent K18-hACE2 mice were at or below limit of detection against XBB.1.5 [GMT 10, IQR 0.00]. Likewise, XBB.1.5-convalescent nAb titers against WA1 [GMT 11.33, IQR 47.36] were at or below the limit of detection. These results confirmed that mice infected with the ancestral or a contemporary variant of SARS-CoV-2 lacked nAbs against the antigenically distinct variant.

**Fig. 2:**
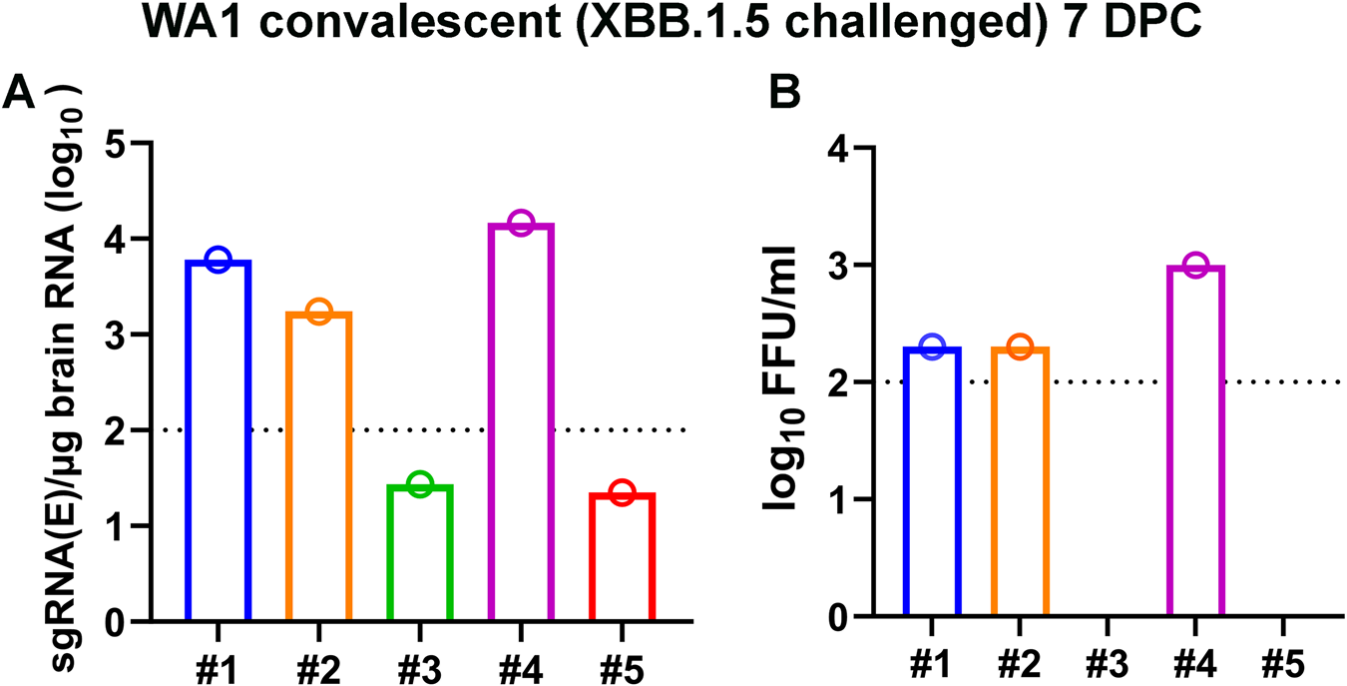
Presence of SARS-CoV-2 virus in K18-hACE2 mice that succumbed to infections. **A** Viral sgRNA levels in brains of WA1 convalescent (XBB.1.5 challenged) mice at 7 DPC were quantified by qRT-PCR. **B** Infectious virus from brain lysates was determined by focus-forming assays (limit of quantification is 100). Numbers 1–5 in **A** and **B** denote individual animals in the group.

Two months after the initial infection, we challenged convalescent and age-matched naive mice with 10^5^ PFU WA1 or XBB.1.5 to examine cross-protection in the absence of nAbs (Fig. 1C–F). Six groups of mice were included: naïve mice challenged with WA1 (*n* = 5); naïve mice challenged with XBB.1.5 (*n* = 10); WA1 convalescent mice challenged with WA1 (*n* = 5), WA1 convalescent mice challenged with XBB.1.5 (n = 10); XBB.1.5 convalescent mice challenged with WA1 (*n* = 9), and XBB.1.5 convalescent mice challenged with XBB.1.5 (*n* = 5). After the challenge, 4–5 mice in three groups (naïve mice challenged with XBB.1.5, WA1 convalescent mice challenged with XBB.1.5, and XBB.1.5 convalescent mice challenged with WA1) were monitored for weight loss and survival. Another five mice from each of the six groups were euthanized at 3 days post-challenge (DPC) for viral load analyses. Notably, some convalescent mice that were challenged with the heterologous virus started losing weight on 5 DPC with 50% of XBB.1.5 (2 out of 4) and 20% of WA1 convalescent mice (1 out of 5) succumbing to the heterologous challenge by 9 DPC (Fig. 1C, D). To confirm brain infection in convalescent mice, we repeated the challenge study with XBB.1.5 in 5 WA1 convalescent mice. At 7 DPC, both viral subgenomic RNA (sgRNA) and infectious virus were detectable in brain homogenates in 3 out of the 5 (60%) mice (Fig. 2).

Convalescent mice largely had undetectable viral load in the lungs at 3 DPC, except one mouse from the WA1 convalescent group challenged with WA1 or XBB.1.5 (Fig. 1E, F). Undetectable viral load levels were also observed in bronchoalveolar lavage fluid (BALF) collected from convalescent mice (Fig. 1E, F). Sporadic infectious viral load was detected in nasal turbinates of convalescent mice, but the levels remained significantly lower than that from the naïve mice after challenge (XBB.1.5 convalescent mice challenged with WA1 vs. naïve mice challenged with WA1, *p* < 0.05; XBB.1.5 convalescent mice challenged with XBB.1.5 vs. naïve mice challenged with XBB.1.5, *p* = 0.002; WA1 convalescent mice challenged with XBB.1.5 vs. naïve mice challenged with XBB.1.5, *p* < 0.05; WA1 convalescent mice challenged with WA1 vs. naive mice challenged with WA1, *p* < 0.05). Notably, convalescent mice that received the heterologous challenge virus demonstrated similar viral loads in the lung compared to convalescent mice that received the homologous challenge, despite the absence of nAbs for the challenging strain. These results indicate that natural immunity obtained from previous infection remains cross-protective in the lower respiratory tract against challenge with a distant SARS-CoV-2 variant.

**Fig. 3:**
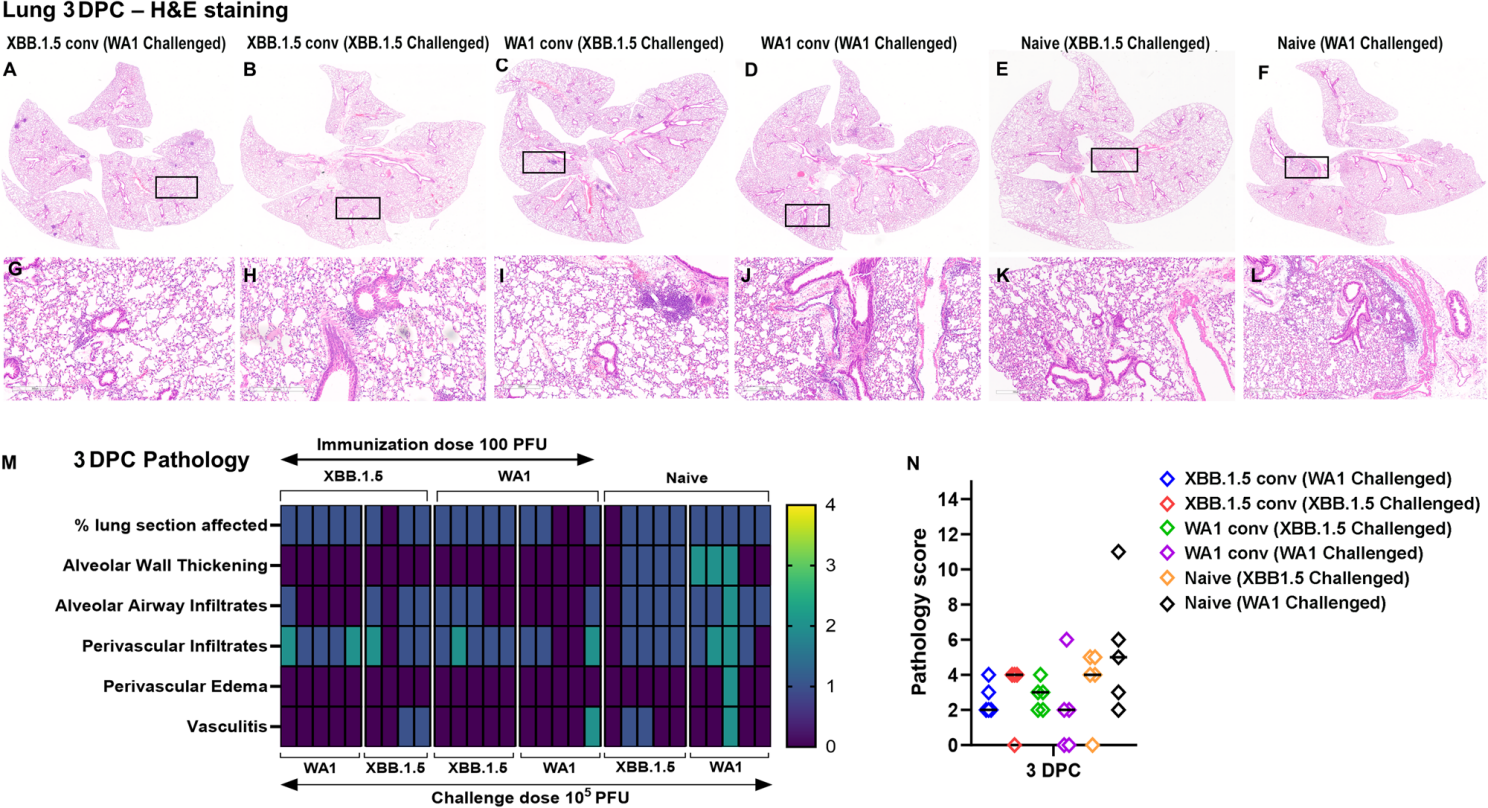
Examination of histopathology corroborates cross protection of convalescent K18-hACE2 mice against homologous or heterologous virus challenges. **A**–**L** Representative images of H&E-stained lung tissue sections (5 µm thickness) collected at 3 DPC. **A**, **G** XBB.1.5 convalescent (WA1 challenged), **B**, **H** XBB.1.5 convalescent (XBB.1.5 challenged), **C**, **I** WA1 convalescent (XBB.1.5 challenged), **D**, **J** WA1 convalescent (WA1 challenged), **E**, **K** naïve (XBB.1.5 challenged), **F**, **L** naïve (WA1 challenged). Black rectangular boxes in **A**–**E** indicate regions from which magnified images in **G**–**L** are taken. Scale bars in **A**–**F** 4 mm and 0.6x magnification, **G**–**L** 300 µm & 8.8x magnification. **M** Heatmap presentation of histopathology scores of lungs collected at 3 DPC based on each category (refer method section for scoring criteria). **N** Cumulative histopathology scores of infected lungs at 3 DPC (*n* = 5 mice in all groups except (*n* = 4) in XBB.1.5 convalescent (XBB.1.5 challenged) group. Bars in the scatter dot plot indicate median values.

**Fig. 4:**
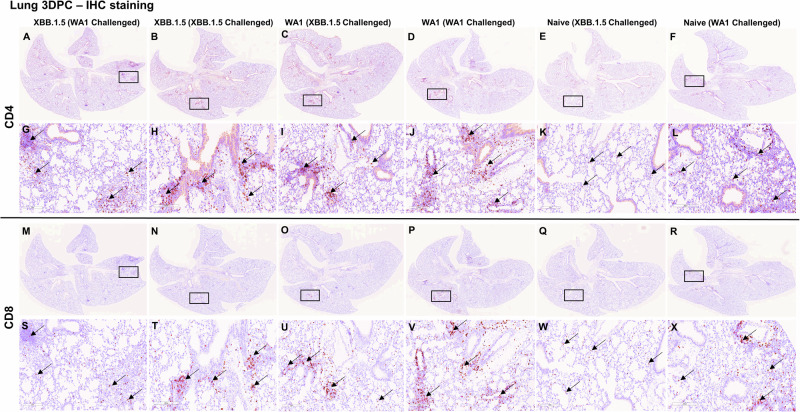
Immune activation in the lungs. Lung tissue sections from 3 days post challenged mice were subjected to immunohistochemistry staining for CD4 and CD8 T cell markers. **A**–**L** represent CD4 T cell marker staining and **M**–**X** represent CD8 T cell marker staining. **A**, **G**, **M**, **S** XBB.1.5 convalescent (WA1 challenged), **B**, **H**, **N**, **T** XBB.1.5 convalescent (XBB.1.5 challenged), **C**, **I**, **O**, **U** WA1 convalescent (XBB.1.5 challenged), **D**, **J**, **P**, **V** WA1 convalescent (WA1 challenged), **E**, **K**, **Q**, **W** naïve mice XBB.1.5 challenged, **F**, **L**, **R**, **X** naïve mice WA1 challenged. Black rectangular boxes indicate magnified regions. **G**–**L** are closeup images of **A**–**F**, and **S**–**X** are closeup images of **M**, **N**, **O**, **P**, **Q**, **R**, respectively. Black arrows that point to brown staining in panels **G**–**L** indicate CD4 positive staining and in **S**–**X** indicate CD8 positive staining. Scale bars in **A**–**F** & **M**–**R**) 4 mm and 0.6x magnification, **G**–**L**, **S**–**X**) 200 µm & 13.2x magnification.

We subsequently performed hematoxylin and eosin (H&E) staining on lung tissues collected at 3 DPC. Histopathology was found to scale with viral loads (Fig. 3A–L). While naïve mice developed observable pathologies, including alveolar wall thickening and alveolar airway infiltrates in the lungs after virus challenge, convalescent mice showed fewer histological changes after challenge with either a homologous or a heterologous virus (Fig. 3M, N). We also assessed immune activation by immunohistochemistry and found CD4^+^ and CD8^+^T cells in the lungs of all groups at 3 DPC (Fig. 4).

### Cross protectivity conferred by candidate live attenuated vaccines

**Fig. 5:**
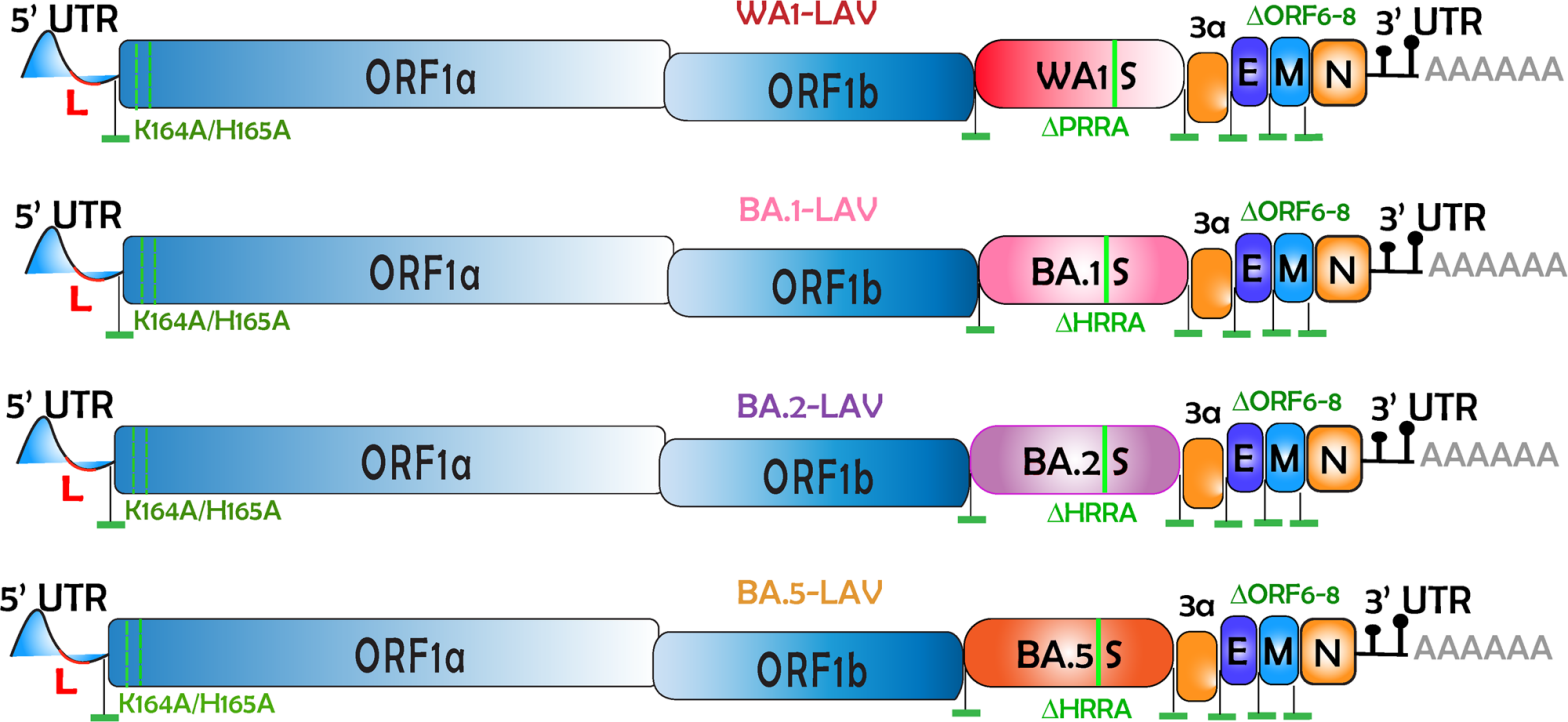
Genome organization of WA1-LAV, BA.1-LAV, BA.2-LAV, and BA.5-LAV. Leader sequence (red), transcriptional regulatory sequence within the leader sequence, and within the body are highlighted as green bars. The polybasic insert “PRRA” or “HRRA” was removed from the spike protein proteins and ORF6-8 were removed from the WA1/2020 backbone. Locations of K164A/H165A within Nsp1 are highlighted in the figure. These viruses are 100–1000 times attenuated compared to the parent strains^31,32^.

**Fig. 6:**
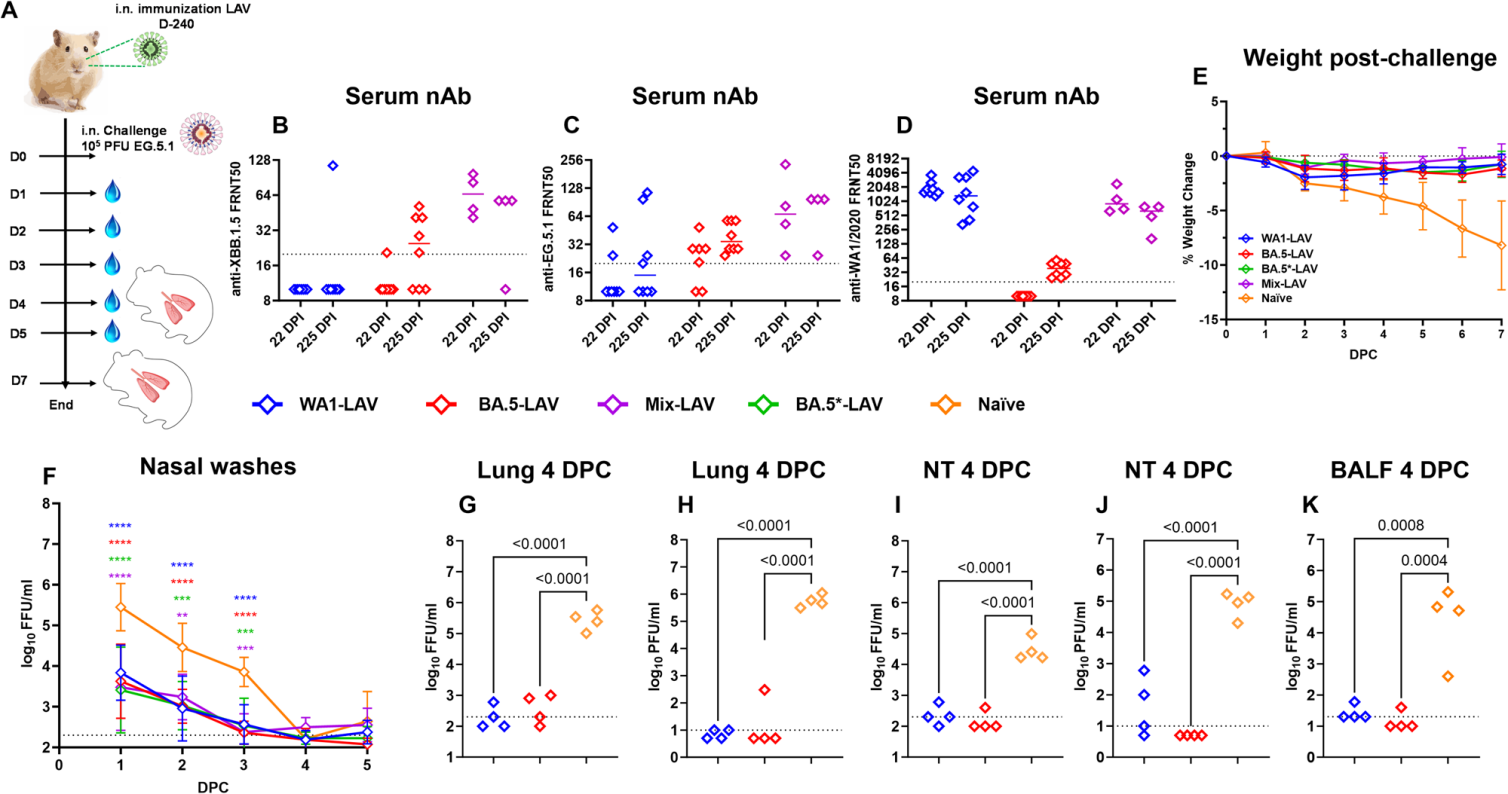
Vaccinations of LAV candidates protect Syrian hamster against EG.5.1 challenge. **A** Overall design of the study. Three-month-old male Syrian hamsters were intranasally inoculated with LAVs (100 PFU WA1-LAV, blue, *n* = 8; or BA.5-LAV, red, *n* = 8; or 100 PFU concoction mixture [25 PFU WA1-LAV + 25 PFU BA.1-LAV + 25 PFU BA.2-LAV + 25 PFU BA.5-LAV, violet, *n* = 4]; or 10^4^ PFU BA.5-LAV (BA.5*-LAV), green, *n* = 4; or PBS (naïve group, orange, *n* = 8). Animals were bled at 22- and 225-days post immunization to collect sera and tested for circulating neutralizing antibodies against XBB.1.5 (**B**), EG.5.1 (**C**) and WA1 (**D**) by FRNT_50_ assay. Bars in the scatter dot plots indicate median values. **E** Weight change recorded for 7 DPC with 10^5^ PFU EG.5.1. **F** Nasal wash viral titer detected by focus-forming assay (p**=0.0018, p***=0.0002, p****<0.0001; 2-way ANOVA Dunnett’s multiple comparison test). Infectious viral titers from lung homogenates, nasal turbinates, and BALF at 4 DPC were determined by focus-forming assay (**G,** **I,** **K**) or plaque-forming assay (**H,** **J**); *n* = 4 animals in each group.

Our laboratory has previously designed multiple LAV candidates in which three attenuating modifications were introduced into the WA1 genome: the removal of the furin cleavage site, the deletion of ORFs 6–8, and introduction of a pair of mutations to the Nsp1 protein^21^. To determine if a vaccine designed against an ancestral virus may protect against contemporary variants, we tested our LAVs in Syrian hamsters, which are highly susceptible to SARS-CoV-2 and have been widely used in COVID-19 research^21–24^. Besides the prototype WA1-based LAV, we also generated additional attenuated viruses with the WA1 spike replaced with BA.1, BA.2 or BA.5 spike protein called BA.1-LAV, BA.2-LAV, and BA.5-LAV, respectively (Fig. 5). We vaccinated male adult Syrian hamsters with a single, low dose (100 PFU) of LAVs bearing WA1 spike, BA.5 spike or mixed LAV (WA1 + BA.1 + BA.2 + BA.5) (Fig. 6A). At 22- and 225-days post vaccination, serum nAb titers against XBB.1.5 (Fig. 6B), EG.5.1 (Fig. 6C), and WA1 (Fig. 6D) were determined for each hamster by FRNT_50_ assay. As expected, of the WA1-LAV vaccinated hamsters (*n* = 8), only 12.5% (*n* = 1) had nAb titers against XBB.1.5 (GMT 13.5) and 50% (*n* = 4) against EG.5.1 (GMT 21.9) at 225 DPI. Of the BA.5-LAV vaccinated hamsters (*n* = 8), 62.5% (*n* = 5) had detectable anti-XBB.1.5 (GMT 21.8) nAbs and 100% had nAbs against EG.5.1 (GMT 37.9) at 225 DPI. Interestingly, although the BA.5-LAV vaccinated group did not initially have detectable nAbs against WA1, all animals seroconverted by 225 DPI (≥4-fold increase in anti-WA1 nAb titers). Of the LAV-Mix vaccinated hamsters (*n* = 4), all developed nAb antibodies against XBB.1.5 (Fig. 6B) and EG.5.1 (Fig. 6C). Hamsters receiving the mixture of attenuated viruses had the highest geometric mean nAb titers against XBB.1.5 (GMT 63.2) and EG.5.1 (GMT 70.1) at 22 DPI (Fig. 6B, C).

On day 241 after the initial vaccination, WA1-LAV, BA.5-LAV, and Mix-LAV vaccinated hamsters were challenged with 10^5^ PFU of an EG.5.1 isolate. A group of hamsters that had been vaccinated with 10^4^ PFU BA.5-LAV (i.e., BA.5*-LAV group in Fig. 6) were similarly challenged as a control group. Vaccinated hamsters had minimal (<2%) body weight loss over 7 days-post-challenge (Fig. 6E). By contrast, naïve (unvaccinated) hamsters had up to 12% body weight loss at day 7 following challenge. The overall amounts of infectious virus measured from nasal washes were comparable amongst the three vaccinated groups, which were 10–100-fold lower than that from the naïve group at 1 DPC (WA1-LAV, BA5-LAV, BA5*-LAV and Mix-LAV, *p* < 0.0001), 2 DPC (WA1-LAV and BA5-LAV, *p* < 0.0001; BA5*-LAV, *p* = 0.0002 and Mix-LAV, *p* = 0.0018) and 3 DPC (WA1-LAV and BA5-LAV, *p* < 0.0001; BA5*-LAV and Mix-LAV, *p* = 0.0001) (Fig. 6F). Infectious virus titers in the lungs, nasal turbinates, and BALF at 4 DPC were also quantified using focus and plaque forming assay (Fig. 6G–K). Compared to unvaccinated naïve hamsters, all vaccinated animals had 100–1000-fold reduction in viral loads in the lungs (Fig. 6G, H), nasal turbinates (WA1-LAV, BA5-LAV, p < 0.0001) (Fig. 6I, J), and in BALF (WA1-LAV *p* = 0.0008, BA5-LAV *p* = 0.0004) (Fig. 6K). Generally, infectious viral loads from vaccinated hamsters were near, at, or below the limit of qualification of the assay, indicating that a robust protection against a contemporary variant was achieved in the respiratory tract after LAV vaccination.

**Fig. 7:**
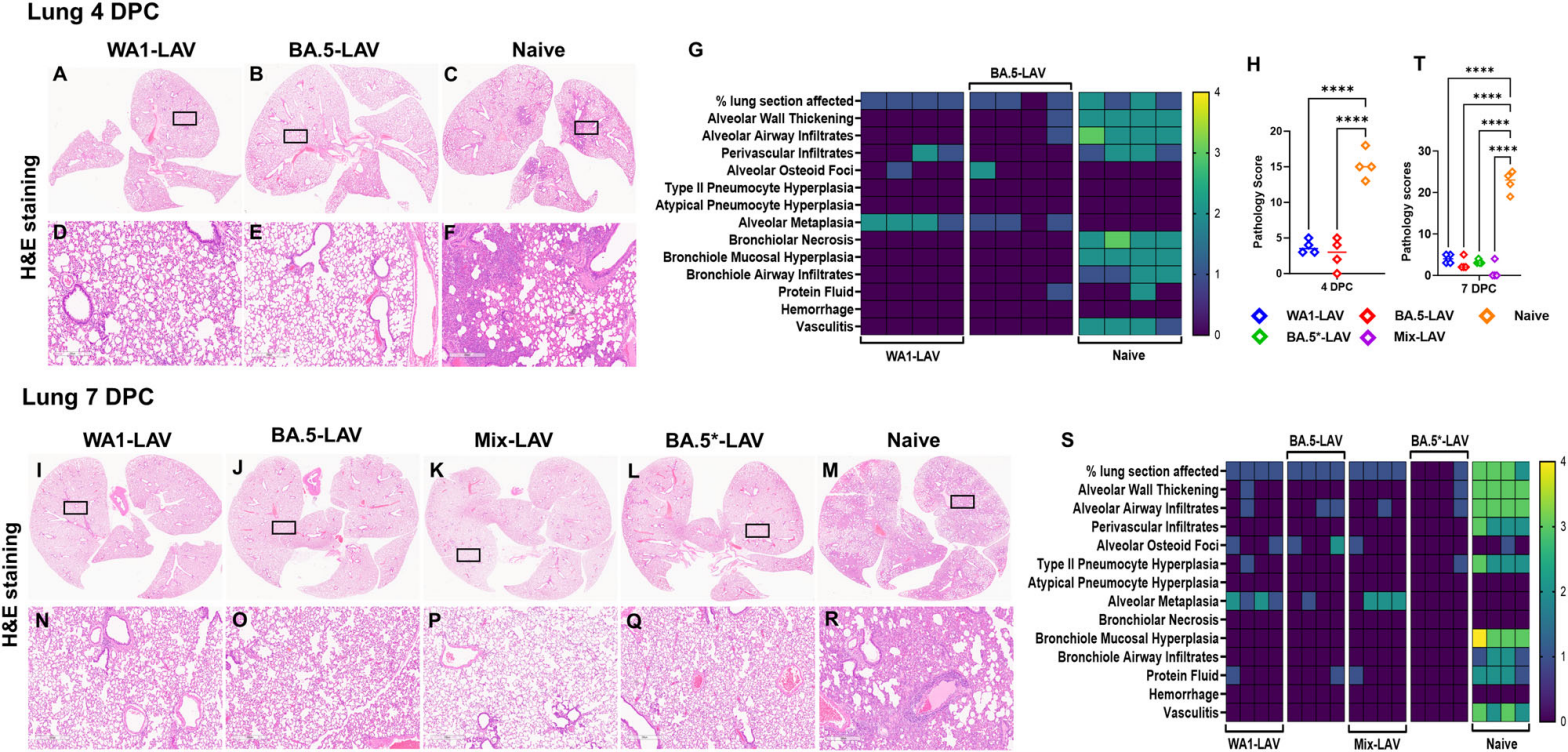
Significant reduction of lung pathology in LAV-vaccinated hamsters upon EG.5.1 challenge. Representative images of H&E-stained lungs from LAV immunized hamsters challenged with 10^5^ PFU EG.5.1 (**A**–**F**) from 4 DPC and **I**–**R** 7 DPC. Rectangular black box in **A**–**C** and **I**–**M** image indicates magnified region. **D**–**F** are closeup images of **A**–**C**, respectively. Similarly, **N**–**R** are closeup image of **I**–**M**, respectively. Scale bar = 6 mm (**A**–**C,** **I**–**M**) and 500 µm (**D**–**F**, **N**–**R**). **G** 4 DPC and **S** 7 DPC heatmap of histopathology scores in the lungs plotted based on each category (refer methods section for criteria details). (**H**) and (**T**) are cumulative lung pathology scores of 4 DPC and 7 DPC. Rhombus symbols in the scatter dot plot: blue (WA1-LAV), red (BA.5-LAV), violet (Mix-LAV), green (BA.5*-LAV) and orange (naïve). The experiment used n = 4 animals per group.

To corroborate the above observation, we also performed H&E staining of the hamster lungs (Fig. 7). For this part of the study, BA.5*-LAV and Mix-LAV vaccinated hamsters (*n* = 4) were euthanized at 7 DPC while animals in the WA1-LAV, BA.5-LAV and naive groups were euthanized at both 4 DPC and 7 DPC (*n* = 4). EG.5.1 infection induced mild to moderate levels of lung consolidation in naïve (unvaccinated) animals (Fig. 7A). Other observable pathologies included alveolar wall thickening, alveolar airway infiltration, perivascular infiltrates, alveolar osteoid foci, bronchiole mucosal hyperplasia, bronchiole airway infiltrates, proteinaceous fluid and vasculitis, bronchiolar necrosis, alveolar metaplasia, and atypical pneumocyte hyperplasia. By contrast, the integrity of alveolar space was preserved among all LAV vaccinated hamster groups after EG.5.1 challenge (Fig. 7A–S). Compared to the naïve group, WA1-LAV, BA.5-LAV and Mix-LAV vaccination reduced total pathology scores with higher dose of BA.5-LAV (BA.5*-LAV group) vaccination being slightly more effective at 7DPC (Fig. 7T). Together, immunization of male Syrian hamsters with our LAV candidates protected against an antigenically distant variant in the lung.

## Discussion

The results of this study are limited since the experiments were conducted in mice and might not be extrapolated to humans. Although the Omicron lineage of SARS-CoV-2 variants might exhibit reduced pathogenicity, the number of hospitalization of patients and cases has not been reduced^25,26^. Additionally, SARS-CoV-2 continues to evolve to escape the population immunity elicited by either natural infection or vaccination. For these reasons, research and preventive measures have been focused on how to improve the available vaccines to combat new variants. Because of so many breakthrough infections in humans, it is postulated that immunity acquired from a previous infection is insufficient to control reinfection with an antigenically distinct variant. We tested such a possibility by infecting K18-hACE2 mice with WA1 or XBB.1.5 and rechallenging the animals with a higher dose of homologous or heterologous virus. Our results show that immunity developed against previous SARS-CoV-2 infection offers protection against reinfection irrespective of SARS-CoV-2 variants in the respiratory tract of K18-hACE2 mice, even in the absence of variant spike-specific neutralizing antibodies. This finding implies that non-neutralizing antibodies and/or cellular immunity effectively offer cross-protection against the tested SARS-CoV-2 variant in our experimental model. Differing from what was observed in the respiratory tract, a fraction of convalescent K18-hACE2 mice still developed viral infection in the brain upon heterologous virus challenge and ultimately succumbed to infection. Such a contrast between the respiratory tract and the brain suggests that neutralizing antibodies may be necessary to block the virus from invading the central nervous system in mice. The lethality of SARS-CoV-2 in K18-hACE2 mice is known to be caused by encephalitis^12–18^, which is rarely observed in humans. It has been reported that administration of aerosolized SARS-CoV-2 to K18-hACE2 mice avoided fatal neuroinvasion^27^, implying that the intranasal route of inoculation could have been the culprit of disproportionately high rates of fatal encephalitis. Indeed, SARS-CoV-2 infections of human brains^28,29^, although recorded, are not common. The often self-reported “brain fog” by some patients is unlikely the result of massive brain infection as observed in the K-18-hACE2 mouse model^30^. For these reasons, the finding that some convalescent K18-hACE2 mice still developed viral infection in the brain upon heterologous virus challenge may not be immediately relevant to human infections.

To expand the above observations into vaccine research, we chose an in-house developed LAV candidate to induce immunity that may be comparable to a natural infection. In this study, we show sustained (~8 months) protection against EG.5.1 challenge in the respiratory tract in Syrian hamsters after vaccination with a LAV based on the ancestral SARS-CoV-2 isolate WA1. Given the significant antigenic difference between WA1 and EG.5.1, our results demonstrate that LAVs can be an attractive addition to the panoply of vaccination strategies in place to combat the ongoing COVID-19 pandemic. Near-universal population immunity to SARS-CoV-2 may somewhat mitigate the safety concerns of using LAV in immune-competent individuals as a booster against emerging variants. In support, we have shown that LAV can boost heterotypic nAbs in previously vaccinated hamsters^31,32^. Similarly, another research group also reported that Syrian hamsters vaccinated with an LAV candidate (sCPD9) developed robust immunity against a heterologous SARS-CoV-2 challenge^33^. This LAV strategy could mitigate concerns over specific antigen-based, vaccine strategies, which can lose protection over time and may lack protection against emerging variants. Notably, mixing LAVs (WA1 + BA.1 + BA.2 + BA.5) induced higher nAB titers against EG.5.1 than did a single LAV, implying that a mixture of antigens might induce a more diverse B cell antibody repertoire, which cross-neutralize a future new variant. It must be pointed out, however, a strong performance of LAV in pre-clinical studies does not guarantee efficacy in vaccine clinical trials. For example, pre-clinical studies have reported superior efficacy of live attenuated influenza vaccine (LAIV) against mismatched influenza viruses, compared to inactivated influenza vaccines^34,35^. Real-world evidence later showed moderate to high efficacy conferred by LAIV against influenza in children but not so much in adults^36–38^. Pre-existing immunity in adults may have dampened the immunogenicity of LAIV^39^.

Due to the constantly evolving nature of SARS-CoV-2, this study was not carried out with the most recent circulating SARS-CoV-2 variant (i.e., LP.8.1 or XEC). The observed brain infection of K18-hACE2 mice during a heterologous virus challenge may not be extrapolated to humans because of the evident anatomic and immunological differences between the two species. Nonetheless, profiling other immune markers in the K18-hACE2 mouse model would likely shed light on how the host manages the burden of immune-evasive variants without specific neutralizing antibodies.

## Materials and Methods

### Cells and Viruses

Vero E6 cell line (Cat # CRL-1586) was purchased from American Type Cell Collection (ATCC) and cultured in Eagle’s minimal essential medium (MEM) supplemented with 10% fetal bovine serum (Invitrogen) and 1% penicillin/streptomycin, and L-glutamine. A549-hACE2 (NR-53821) cells were obtained from BEI Resources and maintained in Dulbecco’s minimal essential medium (DMEM) supplemented with 5% penicillin and streptomycin, and 10% fetal bovine serum at 37 °C with 5% CO_2_. H1299-hACE2 is a human lung carcinoma cell line stably expressing human ACE2. The cell line was generated by lentiviral transduction of the NCI-1299 human lung carcinoma cell line (ATCC CRL-5803) with pLVX-hACE2 and selected with 1 μg/mL puromycin. A western blot was performed to confirm the expression of hACE2. H1299-hACE2 cells were maintained in DMEM supplemented with 5% penicillin and streptomycin, and 10% fetal bovine serum at 37 °C with 5% CO_2_.

The SARS-CoV-2 isolate WA1/2020 (NR-52281, lot 70033175), XBB.1.5 (hCoV-19/USA/MD-HP40900/2022, NR-59105), and EG.5.1 (hCoV-19/USA/MD-HP47946/2023, NR-59576) were obtained from BEI Resources, NIAID, NIH. WA1/2020 had been propagated thrice in Vero cells and once in Vero E6 cells prior to acquisition. It was further passaged once in Vero E6 cells in our lab. The virus has been sequenced and verified to contain no mutation to its original seed virus. XBB.1.5 and EG.5.1 were used immediately upon receipt without subsequent passages. Generation of live attenuated viruses has been described elsewhere^31,32^.

### Mouse infection experiments

Female adult K18-hACE2 mice were previously purchased from the Jackson laboratory and held at the FDA vivarium. All experiments were performed within the biosafety level 3 (BSL-3) suite on the White Oak campus of the U.S. Food and Drug Administration. The study protocol details were approved by the White Oak Consolidated Animal Care and Use Committee and carried out in accordance with the PHS Policy on Humane Care & Use of Laboratory Animals.

For infection studies, mice (7–8 weeks old) were first anesthetized with 3–5% isoflurane. A total of 30 mice were equally split into 2 groups, intranasal inoculation was done by pipetting 100 PFU (WA1 or XBB.1.5) SARS-CoV-2 in 25 µl volume dropwise into the nostrils of the mouse. Mice were weighed and observed daily. After 6 weeks post infection, mice were bled to collect serum samples and stored at −80 °C for future use to determine circulating neutralizing antibody titers against WA1 or XBB.1.5. At day 60 mice were rechallenged with 10^5^ pfu of WA1 or XBB.1.5, were weighed and monitored for any clinical signs of sickness. For tissues and bronchoalveolar lavage fluid (BALF) collection, mice were euthanized by CO_2_ overdose on day 3 postchallenge as necessary.

### Hamster challenge experiments

Male outbred Syrian hamsters (*Mesocricetus auratus*) were previously purchased from Envigo and held at the FDA vivarium. All experiments were performed within the biosafety level 3 (BSL-3) suite on the White Oak campus of the U.S. Food and Drug Administration. The animals were implanted subcutaneously with IPTT-300 transponders (BMDS), randomized, and housed 2 per cage in sealed, individually ventilated rat cages (Allentown). Hamsters were fed irradiated 5P76 (Lab Diet) *ad libitum*, housed on autoclaved aspen chip bedding with reverse osmosis-treated water provided in bottles, and all animals were acclimatized at the BSL3 facility for 4–6 days or more prior to the experiments. The study protocol details were approved by the White Oak Consolidated Animal Care and Use Committee and carried out in accordance with the PHS Policy on Humane Care & Use of Laboratory Animals.

For vaccination studies, three-month-old Syrian hamsters were anesthetized with 3–5% isoflurane following procedures as described previously^40,41^. Hamsters were intranasal inoculated by pipetting 100 PFU of attenuated viruses having WA1 spike or BA.5 spike or 10^4^ PFU of attenuated virus with BA.5 spike or inoculated with 25 PFU each of attenuated viruses having WA1 spike, BA.1 spike, BA.2 spike, BA.5 spike (100 PFU total in the final mixture) in 50 µl volume dropwise into the nostrils of the hamster under anesthesia. Nasal washes were collected by pipetting ~160 µl sterile phosphate-buffered saline into one nostril when hamsters were anesthetized by 3–5% isoflurane. Eight months later, hamsters were challenged with 10^5^ PFU of EG.5.1 in 50 µl volume dropwise into nostrils, were weighed and monitored for clinical signs of sickness. For tissues and BALF collection, a subset of hamsters was humanely euthanized by intraperitoneal injection of pentobarbital at 200 mg/kg, and lungs were harvested for histopathology. Blood collection was performed under anesthesia (3–5% isoflurane) through gingival vein puncture.

### Virus titration

Nasal wash, BALF, and lung homogenate samples were 10-fold serially diluted in 96-well plates and dilutions added to 96-well black-well plates for fluorescent focus forming assays in H1299-hACE2 cells^42^. After 1 hour incubation at 37 °C, a Tragacanth gum overlay (final concentration 0.3%) was added. Cells were incubated at 37 °C and 5% CO_2_ overnight (∼18 h), then fixed with 4% paraformaldehyde, followed by primary staining of cells with rabbit anti-N Wuhan-1 antibody (Genscript U739BGB150-5, 1:2000 dilution) overnight. Cells were then washed three times with PBS 0.1% Tween-20, followed by secondary staining with anti-rabbit Alexa-488 conjugated antibody (1:2000 dilution), and 2 µM Hoechst 33342. The infectious titers were then counted using Gen5 software on a Cytation7 (Agilent) machine and calculated and plotted as focus-forming units per milliliter (FFU/ml). Plaque-forming assays in Vero E6 cells were performed as described previously^31^.

### Real-time PCR assay of SARS-CoV-2 subgenomic RNA

Quantification of SARS-CoV-2 E gene subgenomic mRNA (sgmRNA) was conducted using Luna Universal Probe One-Step RT-qPCR Kit (New England Biolabs) on a Step One Plus Real-Time PCR system (Applied Biosystems). The primer and probe sequences were: SARS2EF: CGATCTCTTGTAGATCTGTTCT; PROBE: FAM-ACACTAGCCATCCTTACTGCGCTTCG- BHQ-1; SARS2ER: ATATTGCAGCAGTACGCACACA. To generate a standard curve, the cDNA of SARS-CoV-2 E gene sgmRNA was cloned into a pCR2.1-TOPO plasmid. The copy number of sgmRNA was calculated by comparing to a standard curve obtained with serial dilutions of the standard plasmid. The limit of quantification (LoQ) of the sgmRNA was determined to be 100 copies/reaction. Values below LoQ were mathematically extrapolated based on the standard curves for graphing purpose. When graphing the results in Prism 10, values below LoQ were arbitrarily set to half of the LoQ values.

### SARS-CoV-2 neutralization assay

Samples were serially diluted 2-fold in 5% FBS DMEM and mixed with 100–200 FFU of SARS-CoV-2 (WA1 or XBB.1.5 or EG.5.1) in a 96-well plate at 37 °C for 1 hour. Sample: virus mixtures were then added to confluent H1299-hACE2 cells in 96-well plates. Cells were infected for 1 h before the inoculum was removed and washed three times with DPBS. A second overlay containing 1.2% Tragacanth gum, 2X MEM, 5% FBS, and DMEM was added to the plate. Cells were incubated at 37 °C for 1 day, then fixed with 4% paraformaldehyde, followed by staining of cells with primary rabbit anti-SARS-CoV-2 N Wuhan-1 antibody (Genscript U739BGB150-5) (1:2000 dilution) overnight, followed by secondary anti-rabbit Alexa-488 conjugated antibody (1:2000 dilution) and Hoechst 33342 staining. Plates were imaged on a Cytation7 (Agilent), and foci were counted using Gen5 software. The 50% endpoint neutralization titers were determined as the reciprocal of the highest dilution providing ≤ half of the number of foci obtained from the negative control well (plain DMEM mixed with 100 PFU virus).

### Histopathology Analyses

Procedures as described previously^40,41^. Both mouse and hamster lung tissues were fixed in 10% neutral buffered formalin overnight and then processed for paraffin embedding. The 5-μm sections were stained with hematoxylin and eosin for histopathological examinations. Images were scanned using an Aperio ImageScope. Blinded samples were graded by a licensed pathologist for the following 14 categories: % lung section affected, alveolar wall thickening, alveolar airway infiltrates, perivascular infiltrates, perivascular edema, alveolar osteoid foci, type II pneumocyte hyperplasia, atypical pneumocyte hyperplasia, alveolar metaplasia, bronchiolar necrosis, mucosal hyperplasia, bronchiole airway infiltrates, proteinaceous fluid, vasculitis. Lesion scoring: 0 = none, 1 = minimal, 2 = mild, 3 = moderate, 4 = severe. A graph was prepared by summing up the scores in each category. The following antibodies were used for immunohistochemistry (IHC) staining: rabbit anti-CD8 alpha (cytotoxic T cell) (#98941S, Cell Signaling Technology, USA) (1:250 dilution) and rabbit anti-CD4 (helper T cell) (#25229S, Cell Signaling Technology, USA) (1:50 dilution).

### Statistical analysis

One-way, two-way ANOVA, or paired t tests were used to calculate statistical significance through GraphPad Prism (10.0.0) software for Windows, GraphPad Software, San Diego, California, USA, www.graphpad.com.

## Data Availability

No datasets were generated or analysed during the current study.
